# Clumping Morphology Influences Virulence Uncoupled from Echinocandin Resistance in Candida glabrata

**DOI:** 10.1128/spectrum.01837-21

**Published:** 2022-02-02

**Authors:** Chenlin Hu, Gary Fong, Sebastian Wurster, Dimitrios P. Kontoyiannis, Nicholas D. Beyda

**Affiliations:** a College of Pharmacy, University of Houston, Houston, Texas, USA; b Chapman University School of Pharmacy, Irvine, California, USA; c MD Anderson Cancer Center, Houston, Texas, USA; d CHI St. Luke’s Health - Baylor St. Luke’s Medical Center, Houston, Texas, USA; University of Minnesota Medical School

**Keywords:** *Candida glabrata*, yeast, echinocandin resistance, morphology, chitin, virulence

## Abstract

Here, we report two paired sets of an index wild-type Candida glabrata bloodstream isolate and subsequent echinocandin-resistant FKS mutant. One paired set exhibited a higher proportion of clumping cells and was more virulent in the invertebrate host Galleria mellonella than the other paired set. No virulence difference between the paired index and FKS strains was observed. These findings imply a potential link of clumping morphology with virulence in C. glabrata that is uncoupled from FKS-mediated echinocandin resistance.

**IMPORTANCE** Candida glabrata is a leading cause of invasive candidiasis. In contrast to other species, it has a high propensity for developing resistance to echinocandins, which are the first-line treatment. Unlike the dimorphic Candida albicans which can grow invasive filamentous hyphae, C. glabrata lacks this ability. Here, we report a link between virulence and clumping cell morphology in two different sets of clinical C. glabrata strains obtained from patients failing echinocandin therapy. One set of paired strains (echinocandin-susceptible and subsequent resistant mutant) had a high proportion of clumping cells in the population and were significantly more virulent than another set which had fewer clumping cells. Additionally, we corroborate that echinocandin resistance does not impart a significant fitness cost. Our findings suggest that clumping morphology may be an important but previously underestimated virulence factor for C. glabrata and also aid our understand for the high prevalence of resistance observed in this species.

## OBSERVATION

Despite the widespread use of echinocandins, acquired resistance remains uncommon in most *Candida* species (∼3%) (1), whereas a relatively high rate of Candida glabrata isolates (>10%) display echinocandin resistance (2–5). The mechanism underlying the propensity of C. glabrata to develop resistance remains unclear. With limited therapeutic options, understanding the biological and morphological alterations associated with C. glabrata echinocandin resistance and their link with pathogenicity is critical.

Large-cell morphologies of fungal pathogens, such as the aggregative phenotype of Candida auris, have been associated with reduced *in vivo* virulence (6). Here, we describe a large clump-like morphology (cells with ≥3 buds) in clinical C. glabrata strains (a wild-type [WT] strain and its echinocandin-resistant FKS mutant) grown in RPMI 1640 liquid medium (RPMI) and study its link with echinocandin resistance and *in vivo* virulence. Specifically, we compared the virulence potential of isolates exhibiting high and low proportions of clump-like cells and studied the impact of echinocandin resistance on the growth rate, cell wall chitin content, morphology, and virulence in two paired index WT strains (A1 and B1) and their isogeneic echinocandin-resistant FKS mutant strains (A2 and B2). Both sets were isolated from two unique hospitalized patients (A and B) according to an institutional review board (IRB)-approved protocol (Committee for the Protection of Human Subjects [CPHS] number 00000044). Both patients had persistent candidemia despite at least 5 days of micafungin (MFG) treatment. The index-WT strains and MFG-resistant strains were isolated from the index blood culture and from a positive blood culture after MFG, respectively.

The MICs of MFG and other antifungals for the WT and resistant strains are summarized in Table S1 in the supplemental material. An analysis of whole-genome shotgun sequencing data showed that paired strains were highly related, with significantly fewer single nucleotide polymorphism (SNP) differences observed between intrapatient (69 to 84 SNPs) versus interpatient (4,580 to 4,644 SNPs) isolates. Both MFG-resistant isolates harbored an FKS2 S663P point mutation as described previously (7). Key features of the two pairs are shown in Table 1. Set A strains (A1 and A2) grew faster in RPMI at 37°C than set B strains (B1 and B2) (Student’s *t* test, df = 10, *P* < 0.00005), whereas growth rates were not significantly different between the paired strains from each patient (Student’s *t* test, df = 4, *P* > 0.05). The cell wall chitin content was analyzed using a previously described calcofluor white (CFW) staining assay with slight modifications (8). The FKS mutants had a higher cell wall chitin content than their corresponding WT strains (A1 versus A2, count_A1_ = 2,247, count_A2_= 2,317, Mann-Whitney U test, *P* < 10^−7^; B1 versus B2, count_B1_ = 2,098, count_B2_= 1,586, Mann-Whitney U test, *P* < 10^−7^), and the observed increase in chitin content could be a compensatory effect of the reduced biosynthesis of β-1,3-glucan in the FKS mutants (9).

**TABLE 1:** *In vitro* features of two pairs of clinical C. glabrata strains

Strain	Patient	FKS genotype	Area of cells with ≥3 buds (μm^2^)				Growth rate (h^−1^)	Doubling time (h)	Chitin^*a*^ (AU)
			Count	Mean ± SD	Min.–max.	Median			
A1	A	WT	918	66 ± 38	37–892	60	0.45 ± 0.02	1.55 ± 0.06	347 ± 154
A2	A	FKS2-S663P	1015	67 ± 39	32–831	61	0.46 ± 0.01	1.50 ± 0.04	445 ± 162
B1	B	WT	1816	77 ± 32	34–469	70	0.39 ± 0.02	1.80 ± 0.08	256 ± 97
B2	B	FKS2-S663P	2622	85 ± 47	36–884	73	0.36 ± 0.02	1.91 ± 0.11	417 ± 165

a Cell wall chitin was analyzed via indirectly measuring the fluorescence intensity of calcofluor white (CFW)-stained C. glabrata, with arbitrary units (AU).

**FIG 1:**
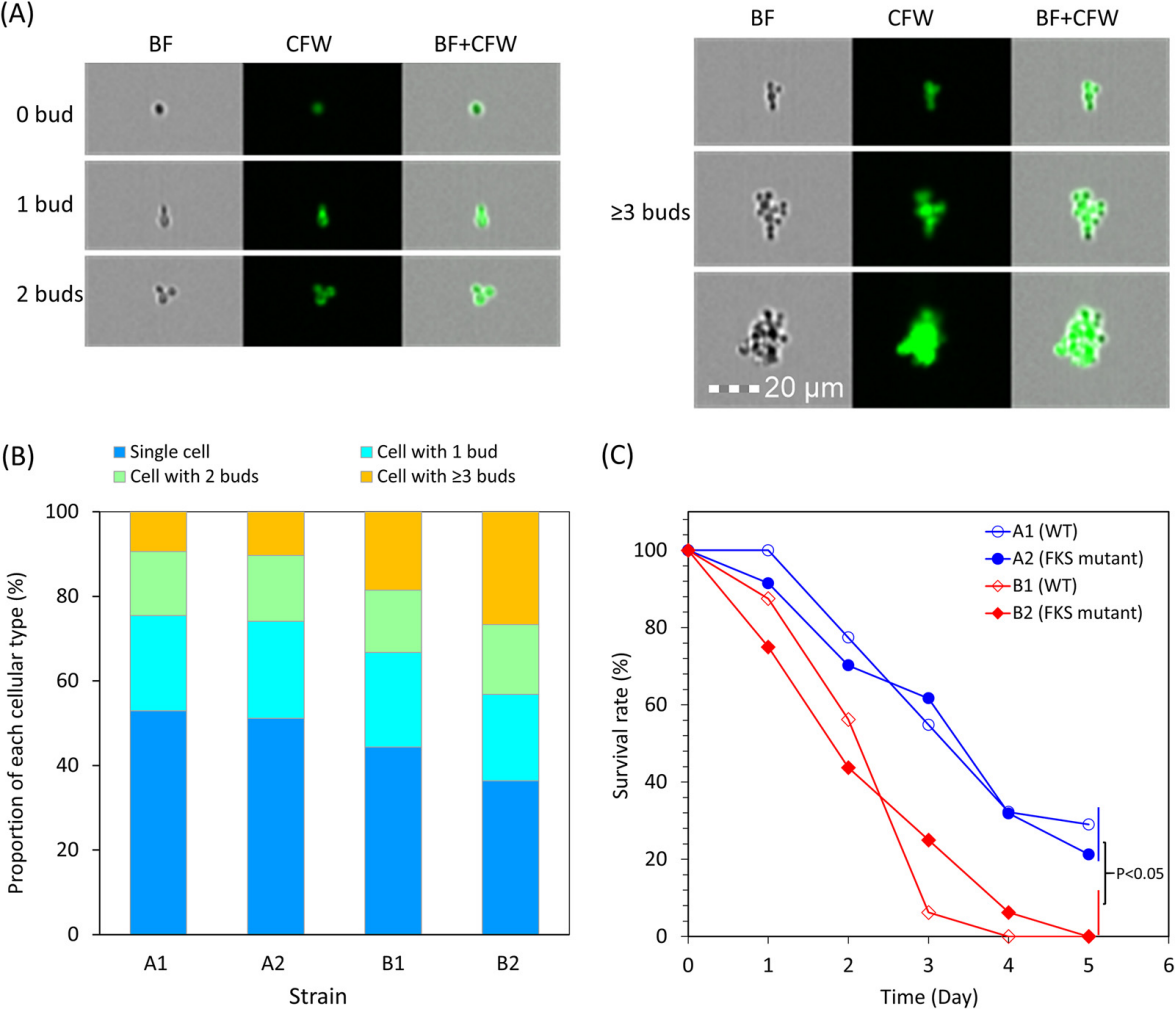
Summary of experimental findings. (A) Representative images of unbudded C. glabrata cells and yeast cells with different numbers of buds (1, 2, and ≥3) obtained using imaging flow cytometry. The yeast cells were stained with calcofluor white (CFW). The brightfield (BF), CFW, and BF+CFW represent the yeast BF image collected in channel 1, the CFW fluorescent image collected in channel 7, and the composite of BF and CFW fluorescent merged images, respectively. (B) Proportion of clumping cells for each C. glabrata strain; each measurement was performed in duplicate. (C) Survival curves of Galleria mellonella infected with each C. glabrata strain.

Using imaging flow cytometry analysis (FlowSight, EMD Millipore-Amins, Seattle, WA), we observed that all C. glabrata strains commonly grew in RPMI mainly as single- and double-bud cells (Fig. 1A). However, the B set strains had a significantly higher proportion of clumped cells (mean, 22.7% ± 5.6%) than the A set strains (mean: 9.9% ± 3.0%) (Student’s *t* test, df = 6, *P* < 0.01) (Fig. 1B). In addition, the median area of clumping cells from the B set strains (71 ± 2.9 μm^2^) was significantly larger than that (60.5 ± 1.3 μm^2^) of the set A strains (Student’s *t* test, df = 6, *P* < 0.001). There was no significant difference in the proportion (or the median area) of clumping cells between the paired strains from the same patient (Student’s *t* test, df = 2, *P* > 0.05). In the sorted clumping-cell populations, the median areas of clumping cells in isolates B1 and B2 were similar (70 versus 73 μm^2^) (Table 1). This result suggests that FKS-mediated echinocandin resistance had a minimal impact on the proportion and size of clumping cells in these two sets of C. glabrata strains.

We subsequently compared the virulence of the two sets of strains using the invertebrate greater wax moth larva (Galleria mellonella) model as described previously with slight modification (10). Briefly, larvae at the final instar stage (*n* = 16,200 to 250 mg of body weight) were injected with 10^7^ cells of each strain suspended in phosphate-buffered saline (PBS), and mortality was monitored for 5 days. Five-day survival rates of larvae infected with strains B1 and B2 were 0%, while 21% to 29% of the larvae infected with strains A1 and A2, respectively, survived until day 5 (Fig. 1C) (*P* < 0.05, log-rank test). In contrast, larvae infected with paired WT and FKS mutant strains did not exhibit significantly different 5-day survival rates (*P* > 0.05, log-rank test), suggesting that acquired echinocandin resistance had a minimal impact on the virulence of C. glabrata in the G. mellonella model.

Taken together, our findings highlight the phenotypic diversity of C. glabrata clinical strains in terms of growth rates, morphology, chitin contents, and virulence but also suggest a potential link of the clump-like morphology and virulence which are uncoupled from echinocandin resistance. The clump-like morphology described here appeared to result from an incomplete separation of daughter cells. Prior research in the phylogenetically related yeast Saccharomyces cerevisiae suggested that clumping hampered cellular growth in the absence of stress but was advantageous in the presence of stress (11). Indeed, despite their growth rate disadvantage (Table 1), the set B strains, which had had a more prominent clumping phenotype, were more virulent in G. mellonella. Thus, the clump-like morphology of C. glabrata could have physiological relevance in adverse environments, such as encounters with host defenses.

The survival advantage of strains growing high proportions of clump-like cells might be explained partially by their improved ability to evade phagocytosis due to the size effect. Previous studies have demonstrated that both the genetic mutation in the chitin synthase gene CHS2 and the deletion of the transcription factor gene ACE2 in C. glabrata resulted in the clumping growth and increased virulence in the corresponding mutants (Evo and Δace2, respectively) (12, 13). Additionally, deletion of the α-(1,2)-mannosyltransferase gene MNN2 resulted in the formation of small cellular aggregates and the increased virulence (14). We detected the single-nucleotide polymorphisms (SNPs) in these genes (CHS2 and ACE2) between two sets of strains (A1 and A2 versus B1 and B2) (see Table S2 in the supplemental material). It would be warranted for future researchers to clarify whether the observed clumping phenotype and virulence are associated with these genes in C. glabrata.

As our study was based on only two sets of C. glabrata isolates, there is a possibility of confounding effects of interstrain variances that are not linked to the clumping phenotype. Further comparative studies using more sets of strains and different *in vivo* platforms would be warranted in order to corroborate the association of clumping morphology with *in vivo* fitness of C. glabrata. In addition, we observed and studied the clump-like morphology of C. glabrata grown in RPMI 1640 medium *in vitro*. Whether the clumping phenotype is found in other growth media and, most importantly, is retained *in vivo* should be addressed in future studies.

Despite these limitations, we describe a multibudded, clump-like phenotype of C. glabrata that might be linked to enhanced fitness and is uncoupled from acquired FKS-mediated echinocandin resistance, contrasting the significant fitness cost of echinocandin resistance observed in C. albicans (15). Such diverging fitness costs between C. glabrata and C. albicans may explain the disparate incidence rates of echinocandin resistance observed between these species.

### Data availability.

The project has been deposited in GenBank under the BioProject numbers PRJNA795464 and PRJNA795468. Accession numbers associated with the whole genome sequences of the isolates were as follows, JAKFQR000000000 (isolate A1), JAKFQU000000000 (isolate A2), JAKFQT000000000 (isolate B1), and JAKFQS000000000 (isolate B2).
