# Supplementary material for: Clumping Morphology Influences Virulence Uncoupled from Echinocandin Resistance in Candida glabrata
Source: Microbiol Spectr. 2022 Feb 2;10(1):e01837-21. doi: 10.1128/spectrum.01837-21 (PMC8809326; doi:10.1128/spectrum.01837-21)
Supplement: SUPPLEMENTAL FILE 1 — Supplemental material. Download SPECTRUM01837-21_Supp_1_seq5.pdf, PDF file, 0.1 MB [file spectrum01837-21_supp_1_seq5.pdf]

Supplemental Materials

Clumping morphology influences virulence uncoupled from echinocandin resistance in *Candida glabrata*

Chenlin Hu[1], Gary Fong[2], Sebastian Wurster[3], Dimitrios P. Kontoyiannis[3],

and Nicholas D. Beyda [1, 4*]

[1]College of Pharmacy, University of Houston, Texas, USA

[2]Chapman University School of Pharmacy, Irvine, CA, USA

[3]MD Anderson Cancer Center, Houston, TX, USA

[4]CHI St. Luke's Health - Baylor St. Luke's Medical Center, Houston, Texas, USA

*Corresponding author: Nicholas D. Beyda (ndbeyda@uh.edu), University of Houston College of Pharmacy, Department of Pharmacy Practice and Translational Research, 4849 Calhoun Road, Houston, Texas 77084 USA

Content:

Supplementary Table S1 and S2

Table S1. Summary of MIC values of two pair of clinical *C. glabrata* strains for 8 common antifungals (micafungin (MFG), caspofungin (CAS), 5-flucytosine (5-FC), amphotericin B (AMB), fluconazole (FLU), itraconazole (ITR), posaconazole (POS), and voriconazole (VOR))

Strain	Patient	FKS-Genotype	MIC (µg/ml)							
			MFG	CAS	5-FC	AMB	FLU	ITR	POS	VOR
A1	A	WT	0.03	0.12	0.06	0.25	64	1	8	1
A2	A	FKS2-S663P	4	8	0.06	0.5	64	16	8	2
B1	B	WT	0.03	0.25	0.06	0.5	16	0.5	1	0.25
B2	B	FKS2-S663P	4	8	0.06	0.5	16	4	1	0.25

Table S2. Summary of single nucleotide polymorphisms (SNPs) analysis of two genes ACE2 (CAGL0M04323g) and CHS2 (GAGL0I04818g) between two sets of strains (A1 and A2 vs. B1 and B2). The sequences of two sets of strains were aligned to that of the reference *C. glabrata* CBS138.

Gene	Strain	Protein effect	Position in CDS codon	Position in CDS	Codon change	Amino acid change
ACE2 (CAGL0M04323g)	B1	Substitution	453	1359	GAG -> GAT	E -> D
	B2	Substitution	453	1359	GAG -> GAT	E -> D
CHS2 (GAGL0I04818g)	A1	Substitution	659	1975	TTG -> ATG	L -> M
	A2	Substitution	659	1975	TTG -> ATG	L -> M
	B1	Substitution	174	520	CAT -> AAT	H -> N
	B2	Substitution	174	520	CAT -> AAT	H -> N
